# Durable Mortar Mixes Using 50% of Activated Volcanic Ash as A Binder

**DOI:** 10.3390/ma18081777

**Published:** 2025-04-13

**Authors:** Andrés Játiva, Andreu Corominas, Miren Etxeberria

**Affiliations:** 1 Department of Civil and Environmental Engineering, Polytechnic University of Catalonia, Jordi Girona 1-3, B1-104A, 08034 Barcelona, Spain; andres.jativa@upc.edu; 2 Department of Architectural Technology, EPSEB—Barcelona School of Building Construction, Polytechnic University of Catalonia, Avenida Doctor Marañon 44, 08028 Barcelona, Spain; a.corominas@upc.edu

**Keywords:** mortar, cement, high-volume volcanic ash, alkali-activated, supplementary cementitous material, calcination, thermal curing

## Abstract

Volcanic ash (VA) is an abundant resource in many world regions that can be used as a supplementary cementitious material (SCM). However, its low reactivity limits its applications as a replacement for Portland cement. In this study, the improvement of its reactivity was evaluated through the calcination of VA (CVA) at 700 °C, alkali activation with Na_2_SiO_3_, CaCl_2_, and Na_2_CO_3_, as well as its combination with other SCMs (lime, fly ash, and blast-furnace slags). Additionally, the effect of curing was analysed under different regimes: standard moist curing and heat curing. The use of alkaline activators, especially 2% Na_2_SiO_3_ and 1% CaCl_2_, along with thermal curing (70 °C for 3 days) in mortars containing 50% VA, resulted in compressive strengths at 28 days, significantly higher than those obtained for mortars with non-activated VA or those cured under moist conditions. Furthermore, the addition of 10% fly ash (FA) and 5% slag (EC) to the mortars also led to the largest improvements in compressive strength. In addition, mortars cured at 70 °C exhibited lower shrinkage and improved resistance to acid attacks, particularly in those manufactured with CVA and 1% CaCl_2_. This study concludes that it is possible to optimise the design of mortars with 50% VA in replacement of ordinary cement based on activation and curing methods. These methods improve early-age strength, reduce shrinkage and water absorption, and enhance acid resistance.

## 1. Introduction

Cement is the second most consumed material in the world after water, and global demand for ordinary Portland cement (OPC) is projected to increase by almost 200% by 2050 [1,2]. However, cement production, the largest industrial CO_2_ emitter, generates 0.6–1.0 tonnes of CO_2_ per tonne of OPC, depending on the manufacturing method used [3], contributing about 8% of global emissions [1]. This substantial carbon footprint highlights the urgent need for sustainable alternatives and innovative approaches to reduce the environmental impact of cement production.

The scientific community has shown a growing interest in better understanding supplementary cementitious materials (SCMs), such as pozzolanic materials of natural or artificial origin, which could be used to partially or fully replace OPC. Given the anticipated shortage of the most common industrial SCMs today and considering their near-universal use in modern cement mixtures, there is an urgent need to identify new SCMs. An effective strategy involves exploring historical materials, such as the use of volcanic ash (VA), which was already employed in Roman times. These materials are valued for their high quality as natural pozzolans, containing high amounts of amorphous silica and alumina [1].

An increase in the pozzolanic activity of VA can counteract the significant weakness of low early-age strength in mixtures containing non-activated VA [4,5]. Consequently, various pre-treatments have been employed to enhance the reactivity of VA [6,7,8,9,10,11]. These treatments are generally classified into three main categories: thermal treatments through calcination, chemical activation methods, and mechanical activations.

The calcination of aluminosilicate materials for activation leads to the loss of volatile components, changes in entropy, reorganisation of atomic structures, and decomposition of crystalline phases; these changes can cause VAs to become more amorphous and reactive [12]. A calcination process above 700 °C is commonly used [11,13,14,15,16] to enhance the reactivity of VA. According to Alraddadi et al. and Askarinejad et al. [13,17], VA subjected to calcination at 700 °C and 800 °C exhibited faster calcium fixation than VA calcined at 900 °C.

VA, composed of aluminosilicate solids, can develop suitable pozzolanic properties when exposed to alkaline conditions induced by an alkaline activator with moderate to high alkalinity [18]. In general, VA particles with high fineness and an alkaline activator solution with a pH of 12–14 promote the dissolution of components and the formation of monomeric aluminate and silicate units, which serve as precursors for aluminosilicate gels [19,20]. Some studies [21,22] have suggested that the amorphous content should be at least 36% to achieve alkali-activated mixtures with adequate properties.

It is well known that VA has lower reactivity than other aluminosilicate materials such as metakaolin (MK), fly ash (FA), and blast-furnace slag (BFS). The addition of reactive materials rich in SiO_2_, Al_2_O_3_, and CaO can help compensate for this deficiency by increasing reactivity and significantly enhancing the properties of the final products [19,22,23,24,25,26]. Furthermore, the incorporation of mineral supplements could replace the thermal curing and calcination process typically used for volcanic ash materials [24].

It is widely known that thermal curing accelerates hydration reactions and improves the early-age strength of cementitious materials. High curing temperatures (i.e., 40 to 90 °C) and prolonged curing periods increase the dissolution rate in the early stages, enhancing the mechanical and physical properties of alkali-activated volcanic ash-based cement [27]. Takeda et al. [28] determined that a curing time of three days was sufficient, as compressive strength did not increase beyond this period. Kani and Allahverdi et al. [29] observed that durability properties also improved due to the formation of additional alkali aluminosilicate gels and the elimination of microcracks.

The activation of VA, as well as the curing method, also influences the physical and mechanical properties of cementitious materials in both fresh and hardened states. The setting time of alkali-activated pastes depends on the particle size distribution and chemical composition of VA materials. Low specific surface area and low free CaO content in the material can prolong the setting times of alkali-activated mixtures [21,30]. Yankwa Djobo et al. [31] demonstrated that VA-activated mixtures cured at 27 °C and 80 °C reached similar density values; however, water absorption and porosity were lower for samples cured at 80 °C than for those cured at 27 °C. This study also found that mortar samples cured at 80 °C exhibited higher compressive strength compared to those cured at 27 °C, even at 90 days.

This study aims to demonstrate the enormous potential of volcanic ash (VA) as an alternative material for replacing 50% OPC. Different VA activation strategies were examined, including thermal activation at 700 °C and alkali activation using various activators (Na_2_SiO_3_, CaCl_2_, and Na_2_CO_3_) at different dosages (1–4%). Additionally, the incorporation of other SCMs (FA, BFS, and lime) as compositional corrective agents for VA was investigated at optimal proportions (5, 10, and 15%), alongside the application of different curing methods (standard wet curing and thermal curing at 40 °C and 70 °C) to identify the most effective combinations for improving mortar performance. The mechanical, physical, microstructural and durability properties of VA-based mortars were assessed. The synergies between the multiple activation techniques enabled enhanced early-age and long-term performances of VA-based mortars while efficiently addressing environmental challenges for cementitious materials.

## 2. Materials and Methods

### 2.1. Materials

All the mortars were produced using high-strength and rapid-hardening Portland cement (CEM I 52.5R, Cement Molins, Barcelona, Spain), an equivalent to ASTM type III Portland cement (C150/C150M-22 Standard Specification for Portland Cement) [32]. The OPC’s chemical composition is shown in Table 1, and Blaine’s specific surface and density were 495 m^2^/kg and 3150 kg/m^3^, respectively. Tap water from the city of Barcelona was also used in all mortar mixes, along with standardised fine aggregate CEN EN 196-1, compliant with ASTM C778 “Specification for Standard Sand” [33]. The fine aggregate’s grain sizes ranged from 0.08 to 2.00 mm. Additionally, a superplasticiser (SP), based on modified polymers in an aqueous solution, was used in the mortars when required.

#### 2.1.1. Volcanic Ash (VA)

The VA used in this study was supplied by a cement factory located in the Otavalo region in northern Ecuador. Otavalo is located in the valley formed by basaltic–andesitic volcanoes Cotacachi and Imbabura. On the southern flank of Cotacachi, the Cuicocha dome partially collapsed, producing pyroclastic flows of block and ash that affected the Otavalo communities around 3500 years ago [34]. After this activity, the volcano entered a brief pause until about 3100 years ago, when it experienced one of the most explosive events to have occurred in the Ecuadorian volcanic arc during the Holocene. It generated major pyroclastic flows and ash falls, with an estimated volume of at least 5 km^3^ [35].

Table 1 shows the chemical composition of the VA, which is mainly composed of silica (SiO_2_) and alumina (Al_2_O_3_). The VA also contained smaller proportions of other pozzolanic oxides, such as ferric oxide (Fe_2_O_3_) and magnesium oxide (MgO). The VA used in this study was similar to the basaltic–andesitic type VA reported in various studies [7,28,36], with silica content ranging from 54.5 to 60% and alumina from 14 to 17% [37]. The sum of the main oxides (SiO_2_ + Al_2_O_3_ + Fe_2_O_3_) exceeded 70% (Table 1), which is the minimum percentage required to classify it as a pozzolanic material suitable for mortar production (C618−22 Standard Specification for Coal Fly Ash and Raw or Calcined Natural Pozzolan for Use in Concrete) [38]. The presence of 6.30% CaO in the VA was higher than in other VAs reported [7,21,39,40], which could be favourable, as calcium might be responsible for the cementitious properties of the material and, consequently, for the strength development of the mortars [41,42]. Additionally, the VA exhibited a low loss on ignition (LOI), likely due to the limited content of zeolites or clay-derived minerals, organic matter, and hydroxides [43]. The molar ratio of SiO_2_/Al_2_O_3_ was 3.3, favourable for producing improved properties in alkali-activated materials [44].

According to crystallography and mineral composition defined in previous work [45], VA had 38% amorphous material and was mainly composed of sodium–calcium feldspars or plagioclase (anorthite series) (45.4%). In addition, some minerals, including quartz (4–5%), montmorillonite (2.3%), iron and magnesium oxides, magnetite (7%), and hornblende–magnesium (10%) were also identified in its composition.

The Strength Activity Index (SAI) (ASTM C311-11 Standard Test Methods for Sampling and Testing Fly Ash or Natural Pozzolans for Use in Portland Cement Concrete) was determined and validated to be higher than 75%, which is the minimum required value. The specific surface area of the VA was 1.65 m^2^/g, in line with other studies that showed specific surface areas of VA lower than 2.5 m^2^/g [21,46], and the bulk density of the VA was 2.73 kg/m^3^. The VA was dried at 105 ± 5 °C for 24 h before being used in the production of mortars.

#### 2.1.2. Activator Materials and Treatments

Four activation mechanisms were carried out on the volcanic ashes: thermal activation (calcination of the VA), alkaline activation, the addition of corrective agents, and mixed activation, which consists of the combination of the best previous activation methods.

- Thermal activation (calcination of the VA, CVA)

The mortars with thermal activation were produced with the VA pre-calcined at 700 °C for 1 h (from now on, indicated as CVA), defined as optimum in a previous work [45]. The heating rate was 5 °C for 1 h, followed by rapidly cooling to room temperature (25 °C ± 1). The CVA achieved up to 52% amorphous content, and new minerals emerged, such as forsterite and hematite, which are related to recrystallising minerals rich in iron and magnesium oxides.

- Alkaline activation (AA)

Three types of alkaline activator were used in the mortars: (1) sodium silicate (Na_2_SiO_3_, 99.0% purity, referenced as NaSi, with a composition of 26.4% SiO_2_, 8.0% Na_2_O, 65.5%H_2_O, and a modulus of 3.35); (2) calcium chloride (CaCl_2_, 99.0% purity, powder, referenced as CaCl); and (3) sodium carbonate (Na_2_CO_3_, 99.0% purity, powder, referenced as NaCO).

- Corrective agents

Three corrective agents, fly ash (FA), ground granulated blast-furnace slag (BFS), and powdered lime (L), were selected. The high content of reactive materials, rich in SiO_2_, Al_2_O_3_, and CaO, could help mitigate the limited reactivity of the VA by adjusting the composition of the binder and significantly optimising the characteristics of the final products.

The FA was classified as Class F; the chemical composition of its components is presented in Table 1, and its density was 2.2 kg/dm^3^. The BFS was sourced from an industrial area of Spain, its chemical composition is presented in Table 1, and its bulk density was 2.80 kg/dm^3^. Moreover, powdered lime (Ca(OH)_2_), referenced as L, of type CL-80 S, was used to compensate for the calcium deficiencies of the CVA in the mortar mixtures. Lime’s chemical composition is also shown in Table 1, and its bulk density was 400 kg/m^3^.

### 2.2. Mix Design

Table 2 presents the detailed composition dosage of the mortar mixtures produced with 50% VA in replacement of OPC. In addition, the mortar made with 100% OPC was also produced. In the case of thermal activation, the letter “C” was added before VA to indicate that it was calcinated (CVA50). The mortars produced with AA followed the specific nomenclature: VA50-AA%, where “AA%” denotes the type of activation treatment—NaSi, CaCl, or NaCO. Na_2_SiO_3_ was used in concentrations of 1%, 1.5%, 2%, and 3% with respect to the binder weight (OPC + VA); CaCl_2_ was used in concentrations of 1%, 2%, 3%, and 4%; and Na_2_CO_3_ was used in concentrations of 1%, 2%, and 3%. Moreover, the mortars with 10%, 20%, and 30% of FA and BFS relative to the VA by weight were prepared (5%, 10%, and 15% with respect to the total binder, for example, nominated as VA45FA5, VA35-BFS15, etc.). In addition, a mortar produced, referenced as L, of type CL-80 S was used to compensate for the calcium deficiencies of the CVA in the mortar mixtures, replacing 20% of CVA by weight (10% with respect to the total binder, nominated as CVA40-L10).

In addition, a mixed activation strategy included thermal activation of VA (CVA), alkaline activation by the addition of CaCl_2_ at 1% of the binder weight, and corrective agents by using a 10% replacement of VA by BFS in weight was produced, nominated as CVA45-BFS5-CaCl1.

All mortar mixtures were produced following the ASTM C109/C109M [47], maintaining a binder/fine aggregates (B/FA) weight ratio of 1:2.75 and a constant water/binder (W/B) ratio of 0.48.

Before starting the mixing procedure, the dry components (VA, CVA, OPC, L, BFS, and FA) were thoroughly mixed in the standard bowl of the mechanical mixer. Water was then added to the dry components in the mixing bowl to initiate the mixing process. The bowl was placed in the mechanical mixer, and the process followed the standard procedure as described in the specification. For mortars requiring alkaline activation, the AA was dissolved in the mixing water beforehand, and the solution was slowly added to the dry components during the mixing phase.

After mixing, the mortar flow was measured in the flow table, as specified in the standard (ASTM C1437) [48]. All mortars were produced to obtain similar flowability to the OPC mortar (170 mm ± 5 mm), using SP only on mortars containing NaSi and the mortar CVA40-L10.

After performing the flow test, fresh mortar mixtures were placed in standard 40 mm plastic cube moulds and compacted in a vibrating table in 2 stages of 60 s. Finally, the specimens were wrapped in plastic film and stored in a humidity chamber under conditions of relative humidity (RH) > 95% and a temperature of 22 ± 1 °C for 24 h. After this time, the specimens were demoulded and subjected to different curing processes until the time of testing for their mechanical and physical properties.

### 2.3. Curing Process

Three different curing regimens were carried out to analyse their effects on the hardening properties of the mixtures.

#### 2.3.1. Standard Wet Curing

The specimens were subjected to wet curing in a chamber with a constant temperature of 22 ± 1 °C and a RH of 95%. They were kept under these conditions until the testing age.

#### 2.3.2. Accelerated Thermal Curing at 40 °C with Sealed Samples

Immediately after demoulding, each sample was resealed using plastic film to prevent moisture loss. The sealed samples were placed in an oven at a constant temperature of 40 °C. They remained under these conditions for 3 days to accelerate hydration reactions. After the thermal curing cycle, the plastic film was removed from the samples, and they were transferred to a wet chamber maintained at 22 ± 1 °C and an RH of 95%. The samples stayed under these conditions until reaching the testing age.

#### 2.3.3. Accelerated Thermal Curing at 70 °C with Sealed Samples

The concrete specimens followed the same procedure as in the thermal curing process at 40 °C; however, the oven temperature was raised to 70 °C.

### 2.4. Testing Programme

Two experimental phases were carried out. In Phase 1, the mortars were validated by the compressive strength value assessment at the ages of 7 and 28 days.

The experimental Phase 2 was carried out with the mixtures that achieved superior compressive strength at 28 days in Phase 1. In order to understand the mechanical behaviour, additional tests were performed to assess their properties in both fresh (setting time) and hardened states (mechanical, physical, shrinkage, microstructural, and durability to acid attack).

#### 2.4.1. Phase 1. Test Procedure

- Compressive strength

The compressive strength of the mortars was assessed following the procedure outlined in the ASTM C109/C109 M standard [47]. Compressive strength tests were conducted after 7, 28, and 90 days of curing. The measurements at 90 days were limited to the Phase 2 experimental phase. The testing was performed using a hydraulic press operating at a loading rate of 900 N/s. For each mix design, tests were conducted on three replicates, and the mean values were calculated.

#### 2.4.2. Phase 2. Test Procedure

The compressive strength at 90 days was determined for the mixtures that achieved the superior strength values in Phase 1. In addition, they were produced again to extend their properties in setting time, physical properties, microstructure, drying shrinkage, and acid-attack resistance.

- Setting time

The analysis of the setting time was carried out on mortar pastes by determining the initial and final setting times following the ASTM C191-01 specification [49].

- Physical properties

The physical properties of water absorption, apparent porosity, and density were determined following the ASTM C642-21 specification [50]. Each of these properties was assessed at 7 and 28 days, and the mean value of the three samples was recorded.

- Microstructural properties

The mineralogy and microstructural analysis were carried out using X-ray diffraction (XRD) and Scanning Electron Microscopy (SEM-EDS), respectively. All the mortars were analysed at the same hydration state, as at 28 days after casting, the mortar samples were immersed in acetone for 24 h, followed by vacuum evaporation, and then dried for 3 days in an oven at 40 °C.

The X-ray diffraction (XRD) pattern of the produced mortars, ground which passed through a 50-micron sieve, was obtained using Bruker D8 forward diffractometer operating with cu radiation (CuKα1) and 2θ scanning ranging from 4 to 70° for 30 min. In all mortars, the external parts of the specimens were discarded to avoid areas affected by carbonation when sampling.

A TESCAN CLARA FESEM (TESCAN, Kohoutovice, Czech Republic) microscope was employed for Scanning Electron Microscopy. The analysis was conducted at 20 kV with a working distance (WD) of 20 mm. Energy-dispersive X-ray spectroscopy (EDX) was performed using an Oxford AZtecLive Advanced EDS detector equipped with an Ultim Max 100 mm^2^ SSD detector. The analysis was performed on cubic fragments of approximately 1 cm^3^ without grinding them. Subsequently, the samples were coated with a 5 nm layer of platinum using a LEICA EM ACE600 sputtering system.

- Drying shrinkage

The drying shrinkage values were determined following the ASTM C157 specifications using 25 × 25 × 285 mm^3^ prismatic specimens. The samples were initially cured in a humid chamber at 22 ± 1 °C and 95% RH, sealed within their moulds with plastic film for 24 h. After this period, the samples were demoulded, and their initial length (original length) was measured on the first day as the reference. Following that, they were transferred to a climate-controlled chamber maintained at 20 ± 2 °C and 50% RH, where they remained until the end of the testing period. Changes in the length of the specimens were recorded at 5, 6, 7, 9, 14, 28, 56, and 90 after casting.

Alternatively, the drying shrinkage value of mortars submitted for 3 days at 70 °C curing process was determined. The initial length before and after the curing regime was determined. For the curing regime, the specimens were then wrapped in plastic film and placed in an oven at 70 °C for three days. Upon completion of this period, they were allowed to cool down at laboratory ambient temperature for 15 min before the specimen length was measured on the fourth day after casting. The specimens were then transferred to the climate-controlled after-curing regime chamber (under the same conditions as the others), where they remained until the conclusion of the 90-day study period. The results represent the average values measured in two specimens for each age, expressed in microstrains (με).

- Acid-attack resistance

The acid-attack resistance of specimens was evaluated on 28-day specimens after curing in a humid chamber (including those subjected to thermal curing at 70 °C for the first 3 days). After drying in an oven at 100 °C for 24 h, the specimens were individually immersed in 5% acidic solutions with a pH of < 1. The acidic solutions selected were hydrochloric acid (HCl), sulphuric acid (H_2_SO_4_), and nitric acid (HNO_3_), following the methodology described by Yankwa Djobo et al. [31] and Al-Sodani et al. [51]. This methodology involved weight-loss measurements and residual compressive strength tests after 90 days of exposure to the acidic environment. For this test, triplicate specimens were prepared to evaluate the variation in dry weight after exposure for 90 days. The residual compressive strength was also determined after 90 days of immersion in the acidic medium.

## 3. Results and Discussion: Phase 1

### 3.1. Results: Phase 1

#### Compressive Strength

Table 3, Table 4 and Table 5 show the strength values obtained by the reference mortar (OPC), the control mortar (VA50), and the mortars with activated VA submitted to different curing conditions at 7 and 28 days (Phase 1). As mentioned above, the strength values at 90 days of mortar samples that achieved superior strength at 28 days were analysed. Each mean value is shown next to the corresponding standard deviation.

As expected, all mortars achieved higher strengths in both thermal curing regimes compared to those subjected to standard curing at 7 days after casting, particularly those exposed to 70 °C for 3 days. The control mortar VA50 subjected to 70 °C curing exhibited a 30% higher strength than VA50 cured in a humid chamber, which attained a strength of 25.8 MPa at 7 days. In addition, VA50 curing at 70 °C for 3 days achieved 29.9MPa, 26% higher than the strength of concrete cured under standard conditions.

The mortar produced with calcined volcanic ash, CVA50, achieved higher strength than the VA50 mortar for both 7 and 28 days, regardless of the curing process to which it was subjected. The CVA50 mortar cured in a wet chamber exhibited a 19% and 30% higher strength at 7 and 28 days, respectively, compared to the VA50 mortar. Under thermal curing at 40 °C, the CVA50 mortar achieved 7% and 5% higher strength than the VA50 mortar at 7 and 28 days, respectively. On the other hand, when subjected to curing at 70 °C, the CVA50 mortar showed a 30% and 13% higher strength than the VA50 mortar at 7 and 28 days, respectively. Moreover, the CVA50 mortar showed only a 9% increase after being subjected to a 70 °C curing regime compared to the CVA mortar cured in a humid chamber, which attained a strength of 41.2 MPa.

Among the mortars produced with AA, the mixtures that incorporated CaCl_2_, especially VA50-CaCl1 under curing conditions at 70 °C, showed the highest compressive strength. Under curing conditions at 70 °C, VA50-CaCl1 obtained 39.9 MPa and 47.0 MPa at 7 and 28 days, which was 20% and 18% higher strength than VA50 at 7 and 28 days, respectively. It achieved 12% greater strength than when it was cured in standard conditions, which showed 41.8 MPa at 28 days. In addition, under standard curing conditions, VA50-CaCl1 was the only mortar that achieved a 10% higher strength compared to VA50 at both 7 and 28 days.

On the other hand, the VA50-NaSi2 mortar under standard curing only showed improvements of 5% and 6% compared to VA50 at 7 and 28 days, respectively. For thermal curing, the VA50-NaSi2 mortar cured at 40 °C and 70 °C achieved 10% and 23% higher strength than VA50 at 7 days. However, at 28 days, these improvements were not as significant, with only 4% and 7% higher strength, respectively. However, the VA50-NaSi2 mortar stood out after being subjected to thermal curing at 40 °C and 70 °C, achieving 21% and 27% higher strength, respectively, compared to standard curing, which reached 33.7 MPa.

Based on the experimental results, it can be observed that no VA50 mortar activated with Na_2_CO_3_ improved the strength obtained by the VA50 mortar, regardless of the curing method used.

The use of BFS corrective agents, even at low replacement levels, improved the properties obtained by VA50 in both standard and thermal curing regimes. The VA45-BFS5 achieved 13–22% at 7 days and 17–40% at 28 days higher strengths (in different curing processes) than VA50. For higher replacements, VA40-BFS10 and VA35-BFS15, the improvements compared to VA50 were also slightly larger, ranging from 11 to 33% at 7 days and 12 to 46% at 28 days. In addition, the mortars incorporating BFS (5–10%) achieved a slight increase in strength (1–6%) under thermal curing regimes compared to the strength obtained through standard curing.

Regarding mortars incorporating FA, higher substitution levels than those from BFS were required to achieve significant improvements. Under standard curing conditions, VA40-FA10 and VA35-FA15 mortars exhibited 15% and 17% higher strength than VA50 at 7 days, respectively. At 28 days, VA40-FA10 and VA35-FA15 achieved 22% and 25% higher strengths, respectively. However, under thermal curing at 40 °C, mortars incorporating FA reached only strengths that were 4% and 10% higher than VA50 at 7 and 28 days, respectively. On the other hand, under a curing regime at 70 °C, the VA40-FA10 mortar exhibited 20% and 17% higher strength than the VA50 cured under the same conditions at 7 and 28 days, respectively. In addition, mortars with 5–10% FA additions exhibited significant strength increases (11–57%) after thermal curing compared to standard curing.

In the case of mixed activation, the CVA45-BFS5-CaCl1 mortar at 7 days under standard curing showed a 14% higher strength than that from VA50. Under thermal curing at 40 °C, a similar increase (9%) was achieved, whereas at 70 °C, the improvements were significantly higher (42% compared to VA50). At 28 days, under standard curing, it exhibited up to a 32% higher strength than VA50, reaching the highest strength of 42.8 MPa. Under curing at 70 °C, CVA45-BFS5-CaCl1 attained the highest 28-day strength (50.4 MPa), which was 26% higher than that of VA50 (39.9 MPa). The mixed activation of this mortar, combined with thermal curing at 70 °C for 3 days, achieved a strength equivalent to 96% of the reference OPC mortar (56.6 MPa) cured under wet conditions. The strengths of CVA45-BFS5-CaCl1 cured at 40 °C and 70 °C were 14% and 18% higher, respectively, than the mortar cured in standard conditions, which achieved a strength of 42.8 MPa.

### 3.2. Discussion: Phase 1

According to the obtained results, the positive effect of thermal activation (CVA) aligns with observations reported in previous studies [6,52], where it was highlighted that the calcination process transforms the main components of VA, including minerals and oxides, into amorphous and highly reactive phases. This transformation enhances the reactivity of VA as a binding agent within the mortar matrix, leading to an increase in its mechanical strength.

The AA, based on 1% of CaCl_2_ as an activator, caused the positive effect in accelerating the pozzolanic reactions of VA with OPC, which may be attributed to the increased formation of secondary hydration products, contributing to faster strength development during the early curing stages of the mortar, as described by Juenger et al. [53]. López-Salas and Escalante-García [54] and Kupwade-Patil et al. [41] highlighted that curing at temperatures between 40 and 90 °C enhances the mechanical properties of alkali-activated VA mortars.

The mineral addition of 5–15% to VA mixtures creates a synergetic effect with VA, acting as a thermal catalyst that enhances the activation process at ambient temperature and promotes higher mechanical strength development at early curing ages, aligned with the findings in the literature [24]. However, under thermal curing, specifically at 70 °C, the addition of BFS accelerated the reaction kinetics and activation of VA at early ages, reaching up to 97% of its 28-day strength.

Moreover, the CVA45-BFS5-CaCl1 attained the highest 28-day strength of 50.38 MPa after being cured for 3 days at 70 °C due to the acceleration of pozzolanic reactions of SiO_2_ and Al_2_O_3_ dissolved in an alkaline medium in the presence of 1% CaCl_2_ exposed at early ages up to 70 °C. These factors are crucial for the rapid formation of hydrated calcium silicates and aluminates, leading to increased strength. Previous studies on VA, BFS, and OPC blends highlight that the synergy between these components improves compressive strength by accelerating pozzolanic reactions [55]. Furthermore, alkali activation and thermal curing enhance these reactions, generating more C-S-H and C-A-S-H gels, which increase mechanical strength and play a key role in the solidification and encapsulation of chloride ions in VA-BFS-based mixtures [56,57]. This suggests that chloride presence is beneficial for the formation of essential gels that contribute to the material’s strength and durability.

The thermal curing lost significant influence on strength enhancement at 28 days compared to 7 days; all mortars achieved equal or higher strengths than their standard-cured counterparts, except for VA50-NaSi3, VA35-FA15, VA35-BFS15, and CVA40-L10.

These results suggest that thermal curing is an effective strategy for improving the initial strength of mortars. Additionally, it was observed that the strength of the mortars was generally higher at 70 °C than at 40 °C, which suggests greater activity in the hydration reactions or chemical interactions between the components of the mortar at higher temperatures. Among the alkaline activators, VA50-CaCl1 showed significant strength increases at 7 days when subjected to a thermal curing process, confirming their accelerator effect in the early stages while also maintaining good performance at 28 days. In the case of corrective agents (FA and BFS), it was similarly observed that improvements were significant at 7 days but only remained notable at 28 days for substitutions between 5 and 10%. VA40-FA10 and VA45-BFS5 were found to be the most efficient mixtures. CVA40-L10 showed consistent increases compared to VA50 at 7 and 28 days, especially under standard curing. The mixed activation demonstrated remarkable improvements at both 7 and 28 days, indicating the synergies of its components (BFS and CaCl_2_) under any curing process. CVA45-BFS5-CaCl1 did not show compressive strength growth reductions from 7 to 28 days by the use of thermal curing in the concretes.

## 4. Results and Discussion: Phase 2

In Phase 2, the activated mortars of CVA50, VA50-CaCl1, VA40-FA10, VA45-BFS5, CVA40-L10, and CVA45-BFS5-CaCl1 under standard curing and thermal curing at 70 °C were analysed in comparison to OPC and VA50.

### 4.1. Results: Phase 2

#### 4.1.1. Setting Time

Table 6 shows that the initial and final setting times achieved by the produced mortar mixtures were within the range specified by the standard ASTM C595-14 standard [58], which defined that the initial setting time for Portland cement must be greater than 45 min and should not exceed the final setting time of 420 min.

Regarding the mortars made with 50% VA, it can be observed that the VA50 mortar showed an 18% increase in the initial setting time and an 8% increase in the final setting time compared to the OPC mortar.

Regarding the use of activation strategies, the CVA50 mortar showed a 7% increase in the final setting time compared to the VA50. In contrast, the setting time (both initial and final) of the CVA40-L10 mortar was shorter than that of the CVA50 mortar. The VA40-FA10 mortar showed a 12% increase in setting times compared to VA50, while the VA45-BFS5 mortar showed a reduction of 9% and 5% in initial and final setting times, respectively, compared to VA50. Additionally, it can be seen that the VA50-CaCl1 and CVA45-BFS5-CaCl1 mortars had shorter setting times than VA50 and were similar to OPC mortars.

#### 4.1.2. Dry Density, Water Absorption, and Permeable Pore Volume

Table 7 presents the results obtained for the physical properties of density (ρ), water absorption (WA), and porosity (P) determined at 28 days under the two curing regimes (standard and thermal at 70 °C) studied.

The density of the mortars produced with VA decreased compared to OPC mortar due to the lower density of VA. However, all the activated VA mortars achieved a similar density, with variations of less than 1.5% under standard curing compared to the control mortar, VA50 (2.08 g/cm^3^), where the VA40-FA10 and VA45-BFS5 achieved the highest density in standard curing regime. Mortars cured at 70 °C exhibited densities ranging from 2.06 to 2.11 g/cm^3^. It is worth noticing that the CaCl1-CV50-BFS10 mortar achieved the lowest density, 2.4% less than the VA50 mortar.

Regarding water absorption, while under standard curing, the mortars produced with VA exhibited absorption values ranging from 6.26% to 7.53% (Table 7), and the mortars cured at 70 °C exhibited water absorption values ranging from 5.58% to 8.49%. Under standard curing, the VA50, CVA50, and VA50-CaCl1 mortars showed 6%, 3%, and 5% higher absorption, respectively, compared to OPC mortar (7.1% WA). However, mortars incorporating FA and BFS, specifically VA40-FA10, VA45-BFS5, and CVA45-BFS5-CaCl1, displayed lower absorption than OPC, with reductions of 7%, 9%, and 12%, respectively. Similarly, porosity reductions were observed for mortars containing corrective agents (FA and BFS).

**Figure 1:**
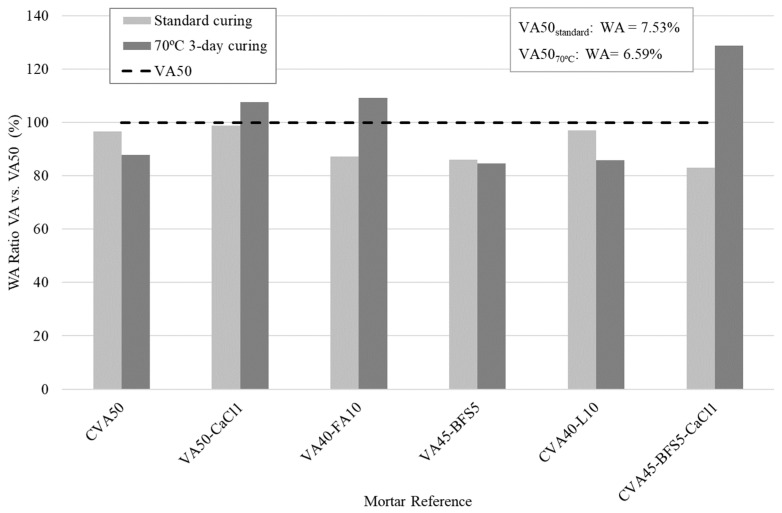
Relative water absorption results at 28 days from those mortars exhibiting superior mechanical performance under standard curing and thermal (70° for 3 days) curing conditions as the ratio of the VA50 under the corresponding curing method.

Figure 1 presents the absorption ratios of each mortar relative to the VA50 (control) mortar at 28 days when the mortars were submitted to standard curing and 70 °C for 3 days curing regimes. According to the standard curing regime, the results indicate that all activated mortars had lower absorption than VA50 (7.53%). Additionally, the CVA45-BFS5-CaCl1 mortar exhibited 16.9% lower absorption than VA50, while the VA45-BFS5 and VA40-FA10 mortars showed reductions of 12.7% and 13.9%, respectively.

#### 4.1.3. Compressive Strength

Table 3 and Table 5 show the compressive strength values obtained at 90 days, indicating that the control mortar, VA50, exhibited the lowest compressive strength among all tested mortars, with 42.59 MPa under standard curing and 45.96 MPa under 70 °C 3-day curing. Compared to VA50, all other mortars showed an increase in strength, with variations ranging from approximately 12% (VA50-CaCl1) to 32% (CVA45-BFS5-CaCl1) under standard curing. The use of CaCl1 (VA50-CaCl1) and BFS (VA45-BFS5) improved strength, particularly under thermal curing. The highest strength gain is observed in CVA45-BFS5-CaCl1, which reaches 56.27 MPa (32% higher than VA50) under standard curing and 51.65 MPa (12% higher) under thermal curing.

As mentioned in Phase 1, thermal curing generally enhances early-age strength, causing a detrimental effect on long-term properties. In the mortars analysed in Phase 2, only VA50 and VA50-CaCl1 achieved higher compressive strength when thermally cured than on the standard curing after 90 days of casting. However, all the VA mortars, VA50, CVA50, VA50-CaCl1, VA40-FA10, VA45-BFS5, CVA40-L10, and CVA45-BFS5-CaCl1, cured under thermal conditions (first 3 days at 70 °C) achieved higher compressive strength at 28 days than those cured under standard conditions. The microstructure analysis was carried out by X-ray diffraction (XRD) and SEM-EDX of mixtures cured under thermal conditions for 28 days.

#### 4.1.4. Microstructure Analysis

- X-ray diffraction (XRD)

**Figure 2:**
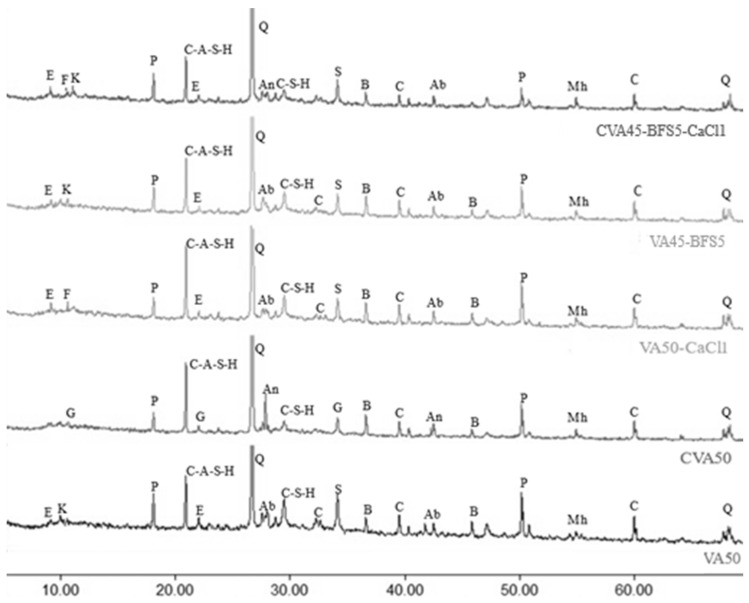
XRD spectrums from mortar mixes VA50, CVA50, VA50-CaCl1, VA45-BFS5, and CVA45-BFS5-CaCl1 under thermal (70° for 3 days) curing conditions at 28 after casting. Legend: Ab: albite; An: anorthite; B: brownmillerite; C: calcite; E: ettringite; F: Friedel’s salt; G: Gismondine; K: kuzelite; Mh: magnesio-horblende; P: portlandite; Q: quartz; S: Strätlingite; C-S-H: calcium silicate hydrate; C-A-S-H: calcium aluminium silicate hydrate.

Figure 2 illustrates the diffraction patterns of the VA50, CVA50, VA50-CaCl1, VA45-BFS5, and CVA45-BFS5-CaCl1 mortars subjected to thermal curing (3 days at 70 °C) at 28 days of age.

The diffractograms reveal the formation of calcium silicate hydrate (C-S-H) and calcium aluminosilicate hydrate (C-A-S-H) gel, attributed to the inclusion of VA, which is rich in aluminosilicate, as gismondine and strätlingite. These components contribute to enhancing both the strength and microstructural properties of the binders. Additionally, all diffractograms identify characteristic mineral phases commonly found in concretes, including quartz, portlandite, calcite, ettringite, kuzelite, and brownmillerite. The albite and anorthite phases, characteristic of VA, are also observed, along with magnesium–hornblende, which is common in andesitic–basaltic VA. Moreover, the mixtures incorporating an alkaline activator (1% CaCl_2_) showed evidence of Friedel’s salt formation.

- Scanning Electron Microscopy (SEM-EDS)

The SEM analysis provided qualitative information on the morphology, combined with EDS analysis, regarding the microstructural characteristics of the produced mortars. The elemental EDS analysis was performed at multiple points on the sample to obtain a real average of the main elements found in each mix.

**Figure 3:**
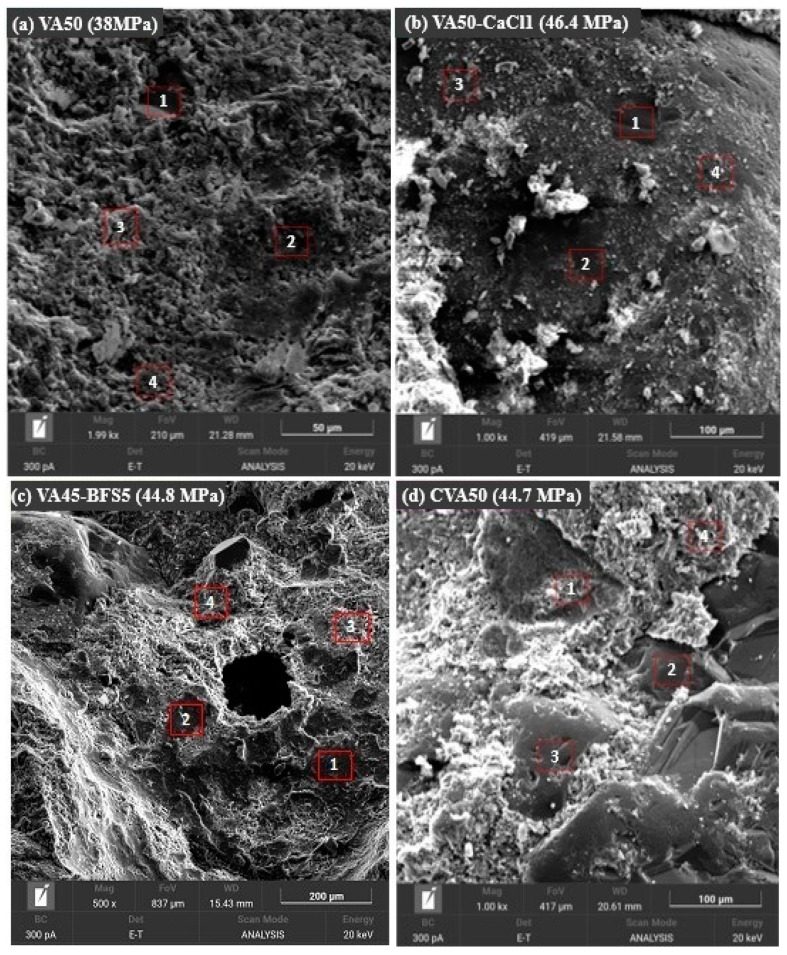
SEM-EDS images from mortar mixes: (**a**) VA50; (**b**) VA50-CaCl1; (**c**) VA45-BFS5; (**d**) CVA50, under thermal (70° for 3 days) curing conditions at 28 after casting.

Figure 3a, corresponding to the VA50 mix, showed a fairly homogeneous and well-networked matrix. The EDS elemental analysis of the selected areas revealed the presence of elements such as Ca, Si, O, Al, S, K, Mg, and Fe. In all areas, the presence of Ca, Al, and Si was indicative of the presence of calcium silicate hydrate (C-S-H) gels from OPC and calcium aluminosilicate hydrate (C-A-S-H), as confirmed by the XRD analysis from this mortar. In zones 2 and 4, sulphur (S) was detected, indicating the presence of the sulphate group, which suggested the presence of ettringite, as also indicated by the XRD. In zone 3, which was rougher and less networked, the presence of Mg, Fe, and K was observed, which could be attributed to unreacted fractions of VA.

In Figure 3b, the VA50-CaCl1 mix showed a more homogeneous matrix with the formation of darker regions throughout the matrix. According to the EDS elemental analysis of zone 1, the presence of Ca and Si indicated the formation of C-S-H. The presence of Al, in addition to Ca and Si, in zones 2 and 4 confirmed the formation of C-A-S-H. On the other hand, zone 3 mainly showed Ca, Al, Cl, S, and O, confirming that these structures were primarily Friedel’s salt or Kuzelite, which was consistent with the phase composition analysed by XRD.

Additionally, in zone 3, the presence of Na, derived from the Na_2_O content in VA, was observed. In an alkaline medium, this may have reacted with the components of the mix. This, along with the presence of Ca, Al, and Si, could indicate the formation of another hydrated gel, N-A-S-H. However, this was contingent on the presence of Ca. If sufficient Ca was present, at a pH above 12, C-A-S-H gel was favoured over N-A-S-H, as observed in the other zones, allowing for the coexistence of both phases, which could precipitate simultaneously. This might lead to the formation of a hybrid (N,C)-A-S-H gel [59,60]. This phenomenon is characteristic of mixes subjected to alkali activation. These gels formed in the system were considered the main reason for the strength growth of this sample.

Figure 3c, corresponding to the VA45-BFS5 mix, showed, according to the EDS elemental analysis at multiple points and based on an average of these, that the sample predominantly contained Ca, Si and Al, indicating the formation of C-S-H and C-A-S-H gels. XRD analysis also confirmed the presence of these gels, along with portlandite and ettringite. All zones exhibited sulphur (S), which was confirmed by the XRD of this sample, showing the presence of ettringite and kuzelite. Additionally, the presence of Na and Mg, originating from the VA, was observed, while the BFS, which contains a higher amount of Ca, also contained Mg and Al. The XRD analysis confirmed the presence of magnesium-containing phases, such as magnesio-hornblende.

Finally, Figure 3d, corresponding to the calcined VA mixture, CVA50, predominantly contained Ca, Si, and Al, indicating the formation of C-S-H and C-A-S-H gels. This was confirmed by XRD, which also identified the presence of portlandite and gismondine.

#### 4.1.5. Drying Shrinkage

**Figure 4:**
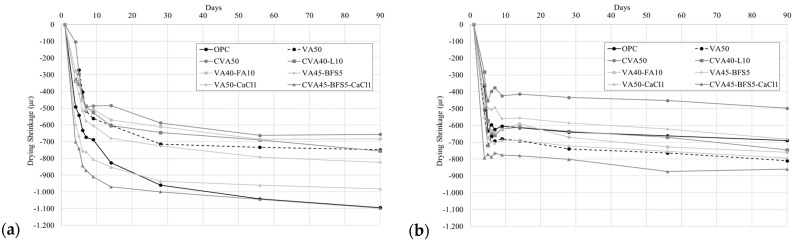
Drying shrinkage results from those mortars exhibiting superior mechanical performance up to 90 days under: (**a**) standard curing and (**b**) thermal (70° for 3 days) curing conditions.

Figure 4 shows the drying shrinkage values achieved by the mortars under standard curing (Figure 4a) and thermal curing at 70 °C for 3 days (Figure 4b), followed by up to 90 days in a climatic chamber at 20 °C and 50% humidity. The highest shrinkage values were observed during the first 4 to 7 days of the test, tending towards much smaller variations after day 9 until the end of the test.

Under standard curing, all mortars made with VA exhibited lower shrinkage than the OPC mortar (−1100 µε). Furthermore, the CVA50 and VA40-FA10 mortars achieved lower shrinkage than the VA50 mortar, with reductions of 12% and 9%, respectively. The mortars with alkaline activation, VA50-CaCl1 and CVA45-BFS5-CaCl1, exhibited greater shrinkage than the reference VA50 mortar, with increases of 31% and 47%, respectively.

After thermal curing, all activated mortars exhibited a lower shrinkage value than that of the VA50 mortar (−810 µε), except for the CVA45-BFS5-CaCl1 mortar, which showed a 6% higher shrinkage value. In addition, the VA50-CaCl1 mortar exhibited a similar behaviour to that of the VA50 mortar. On the other hand, the addition of FA and BFS in the mortars led to a reduction in shrinkage compared to the VA50 mortar, with a 6% reduction for VA40-FA10 and a 26% reduction for VA45-BFS5 (−690 µε, similar to OPC). The CVA50 mortar achieved a lower drying shrinkage value than the VA50 mortar. However, the addition of lime to this mortar (CVA40-L10) resulted in an increase in shrinkage compared to that exhibited by the CVA50 mortar. Nevertheless, both mortars showed lower porosities and water absorptions than VA50.

#### 4.1.6. Acid-Attack Resistance

**Figure 5:**
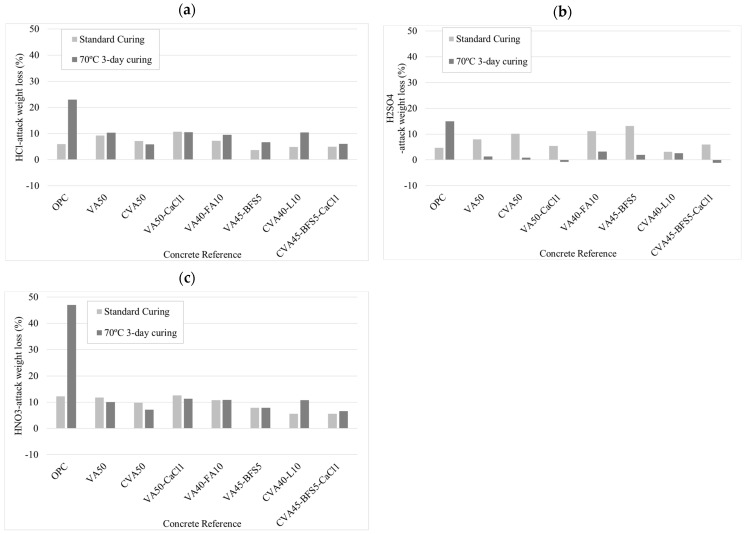
Weight loss results from mortars subjected to acid immersion: (**a**) HCl, (**b**) H_2_SO_4_, and (**c**) HNO_3_.

Figure 5a (mortars immersed in HCl solution) shows that all activated mortar cured in standard conditions experienced less weight loss than that of the control mortar VA50, except for the VA50-CaCl1 mortar, which exhibited an 11% higher weight loss than VA50. The VA45-BFS5 mortar demonstrated the best performance with a weight loss of only 4% after 90 days immersed in acid solution, followed by CVA40-L10, CVA45-BFS5-CaCl, and OPC with a loss of up to 6%. According to mortars cured at 70 °C, CVA40-L10, VA40-FA10, and VA50-CaCl1 mortars described similar behaviour to the VA50 mortar. The VA45-BFS5, CVA50, and CVA45-BFS5-CaCl1 mortars showed lower weight losses of 7%, 6%, and 6%, respectively, compared to the VA50 mortar (10%) after 90 days immersed in acid solution.

Figure 5b (mortars immersed in H_2_SO_4_ solution) shows that VA50-CaCl1, CVA45-BFS5-CaCl1, and CVA40-L10 mortars, cured at standard conditions, achieved 5%, 6%, and 3% weight loss, respectively. In addition, the VA50 mortar lost 8% of its weight, while the CVA50, VA40-FA10, and VA45-BFS5 mortars achieved 10%, 11%, and 13% weight losses, respectively. According to thermally cured samples, while the VA40-FA10 and CVA40-L10 mortars exhibited greater weight loss than the VA50 mortar, the VA50-CaCl1 and CVA45-BFS5-CaCl1 mortars showed lower weight after 90-day immersions.

Figure 5c (mortars immersed in HNO_3_ solution) shows that all activated mortars, cured under standard conditions, exhibited lower weight loss than that of VA50 (12%), except for the VA50-CaCl1 mortar. The mortars with mixed activation, CVA40-L10 and CVA45-BFS5-CaCl1, experienced the lowest weight losses (6%). According to thermally cured samples, the VA50-CaCl1, VA45-FA5, and CVA40-L10 mortars exhibited similar behaviour to the VA50 mortar, with weight losses of 10–11%. On the other hand, the VA45-BFS5, CVA50, and CVA45-BFS5-CaCl1 mortars showed lower weight losses (7–8%).

Table 8 presents the residual compressive strength of each mortar after being submerged in the acidic solution for 90 days. These results should be taken as indicative, as some specimens did not maintain their original dimensions after exposure to the different acidic environments.

The VA50 mortar, under standard conditions, suffered a 34% strength reduction, while the OPC mortar lost 9%. However, the CVA40-L10, VA45-BFS5, and CVA45-BFS5-CaCl1 mortars obtained lower strength losses (5–8%) than that of OPC, in line with the mass loss (see Figure 5). Regarding exposure to H_2_SO_4_, CVA40-L10 and CVA45-BFS5-CaCl1 were the only mortars that exhibited lower strength reductions than VA50 (58%). In response to HNO_3_ exposure, all mortars experienced significant strength losses (approximately 40–70%), except for CVA40-L10 and CVA45-BFS5-CaCl1, which showed much lower compressive strength reductions (20%).

In mixtures cured in thermal conditions, the CVA50, VA45-BFS5 and CVA-BFS5-CaCl1 suffered a decrease below 30% (see Table 8) compared to their pre-attack strength (see Table 3, Table 4 and Table 5), whereas the VA50 mortar exhibited a strength loss exceeding 50% with respect to its initial value. For mortars exposed to H_2_SO_4_, the CVA40-L10 and CVA45-BFS5-CaCl1 mortars exhibited strength losses of 10% and 30%, respectively, whereas the VA50 mortar lost over 50% of its strength. Regarding exposure to HNO_3_, although all mortars experienced significant strength losses (above 40%), all activated mixtures suffered less reduction than the VA50 mortar. The CVA50, CVA40-L10, and CVA45-BFS5-CaCl1 mortars exhibited losses below 50%, whereas the VA50 mortar experienced a 70% strength reduction.

### 4.2. Discussion: Phase 2

#### 4.2.1. Setting Time

The mortars produced using 50% VA in replacement of OPC obtained higher setting time than the OPC mixtures. Meddah et al. [61] justify this increase in setting time by the low reactivity at early ages in mortars made with VA compared to OPC mortars. On the other hand, Al-Fadal et al. [62] observed significant increases in initial setting times in mortars with up to 40% VA replacement in cement pastes, with an increase of up to 48% in setting time compared to OPC mortars.

However, the use of the AA procedure reduced the setting time of VA mortars. Specifically, using the CaCl_2_ activator, the VA50-CaCl1 and CVA45-BFS5-CaCl1 mortars achieved the shortest setting times and were similar to OPC mortar. CaCl_2_ is one of the most recognised and effective accelerators of hydration, setting, and early strength development in Portland cement. The accelerative power of CaCl_2_ is due to its ability to flocculate C-S-H, facilitating the diffusion of ions and water through the initial C-S-H forms [53]. In addition, using BFS, the VA45-BFS5 achieved a similar setting time OPC. However, as mentioned above, all the mortars produced using 50% of VA achieved acceptable setting times.

#### 4.2.2. Physical Properties

Based on density results, although the VA50 produced using 10% FA and 5% BFS obtained the highest density, 2.11 g/cm^3^, in the standard curing regime, the curing methods had minimal influence, as the density of the VA50 mortars cured in thermal conditions (for 3 days at 70 °C) varied less than 1.5% compared to that the same VA50 mortars under standard curing.

Similarly to density, under standard curing, the WA of the mortars produced using FA and BSF, VA40-FA10, VA45-BFS5, and CVA45-BFS5-CaCl1 was lower than the VA50 mortar. The addition of FA and BFS positively affected the physical properties of VA mortars, aligning with the findings of Ren et al. [63], who explained that FA drops porosity and water absorption due to its finer particle size, which reduces interconnected voids. Additionally, the use of BFS as an SCM has also been previously associated with improvements in porosity [64].

Figure 1 indicates that, unlike standard curing, not all the activated mortars reduced their absorption compared to VA50 when the thermal cured regime was used. The CVA50, CVA40-L10, and VA45-BFS5 mortars achieved absorption reductions between 12.1% and 15.3% relative to VA50, whereas the VA40-FA10, VA50-CaCl1, and CVA45-BFS5-CaCl1 mortars showed higher absorption than VA50. The CVA45-BFS5-CaCl1 mortar, in particular, exhibited 29% higher absorption, contrasting with the standard curing condition, where the opposite trend was observed. Regarding curing conditions, water absorption generally decreased when mortars were cured at 70 °C compared to wet curing, except for the mixtures containing FA or the combination of BFS and CaCl_2_. This phenomenon may be related to changes in the porous structure of the mortars due to the curing temperature.

#### 4.2.3. Compressive Strength

In Phase 2, only VA50 and VA50-CaCl1 mixtures that were thermally cured achieved higher compressive strength than on the standard curing after 90 days of casting. However, all the VA mortars, VA50, CVA50, VA50-CaCl1, VA40-FA10, VA45-BFS5, CVA40-L10, and CVA45-BFS5-CaCl1, cured under thermal condition (first 3 days at 70 °C) achieved higher compressive strength at 28 days than those cured under standard condition, due to their microstructure development.

#### 4.2.4. Microstructure Analysis

The SEM-EDS analysis of VA50 mortar showed the presence of Ca, Al, and Si, which was indicative of the presence of calcium silicate hydrate (C-S-H) gels from OPC and calcium aluminosilicate hydrate (C-A-S-H), as confirmed by the XRD analysis from this mortar. The XRD analyses also revealed the presence of gismondine and strätlingite, which have been identified as products of pozzolanic reactions arising from the interaction between SiO_2_ and Al_2_O_3_ from VA with CaO from OPC in the presence of water [65]. Both gismondine and strätlingite contribute to optimal densification and compaction of the mortar matrix, enhancing the overall performance and long-term durability of these materials [66].

Gismondine readily forms at temperatures close to 85 °C and can persist at lower temperatures in most cementitious systems with a high aluminosilicate content [66,67]. Cementitious matrices based on OPC and pozzolans with high amorphous alumina content, when subjected to high curing temperatures, have been shown to primarily promote the formation of gismondine [68]. These findings support the exclusive presence of this phase in the CVA50 mixture, where the high calcination temperature increased the amorphous aluminosilicate content, and the curing conditions at elevated temperatures facilitated its crystallisation.

On the other hand, although strätlingite can be a potential precursor of gismondine, it predominantly crystallises in systems with a high calcium and alumina content, remaining stable at temperatures up to 70 °C. This phase was observed in systems such as VA50-CaCl1, VA45-BFS5, and CVA45-BFS5-CaCl1, which are rich in calcium and alumina. In these systems, Ca^2^⁺ ions promote the formation of calcium-rich C-S-H bonds, and together with silica and alumina, they facilitate the development of phases such as C-A-S-H and strätlingite [67,68]. Some studies have confirmed the presence of strätlingite in materials containing BFS, suggesting the influence of this component on its occurrence [63,68,69]. In the case of the VA50 mixture, which contains lower amounts of alumina and calcium compared to the other mixes, the stability of strätlingite is attributed to the presence of kuzelite and ettringite [69]. Specifically, in the CVA-BFS-CaCl1 mixture, although CVA could promote the presence of gismondine, the coexistence of the activator and BFS determines that the predominant phase is strätlingite.

SEM-EDS indicated matching compositions with Friedel’s salt and Kuzelite on the mixtures incorporating an alkaline activator (1% CaCl_2_). XRD showed evidence of Friedel’s salt formation in VA50-CaCl1 and both in CVA45-BFS5-CaCl1. Other studies [70,71,72] found that such salts can coexist due to the ability of Cl⁻ to bond with materials rich in Al and Ca. In AA mortars, the VA acted as an additional source of aluminium and Cl⁻ bonded, forming Friedel’s salt, as XRD analysis indicated.

The presence of Friedel’s salt may indicate the potential impact of chloride ions in these mixtures, contributing to the enhanced strength of these mortars [73,74], as evidenced by the high-strength values obtained. Additionally, Friedel’s salt plays a crucial role in increasing the durability of concrete by facilitating the immobilisation of chloride ions within its structure, thereby reducing the risk of reinforcement steel corrosion [70,75]. However, it is important to note that the stability of this phase may decrease with rising temperatures, as high temperatures can lead to the decomposition of Friedel’s salt, affecting the matrix’s ability to retain chlorides. This phenomenon could explain the increase in porosity observed in the CVA45-BFS5-CaCl1 mixture under thermal curing compared to the same mixture under wet curing. Nevertheless, its 28-day strength was not negatively affected. Another common phenomenon found in AA mortars is the formation of a hybrid (N,C)-A-S-H gel [59,60]. The presence of Na, found in SEM-EDS results, leads to the formation of additional hybrid gels that enhanced strength growth in these AA mortars.

#### 4.2.5. Drying Shrinkage

Under standard curing, the shrinkage values at 90 days ranged from 650 με for the CVA50 mortar to 1100 με for the CVA45-BFS5-CaCl1 and OPC mortars. Itim et al. [76] described similar behaviour when studying shrinkage for OPC replacements ranging from 10% to 30% with natural pozzolana. They found that, with increasing replacement amounts, shrinkage decreased to values below the OPC (1250 με) over a 6-month period. Furthermore, the AA mortars, VA50-CaCl1 and CVA45-BFS5-CaCl1, along with the OPC mortar, presented the highest shrinkage values under this type of curing. Matalkah et al. [77] also concluded that the shrinkage of alkali-activated mortars was higher.

After thermal curing, the shrinkage values at 90 days ranged from 500 με for the CVA50 mortar to 810 με for VA50 and reached 860 με for the CVA45-BFS5-CaCl1 mortar. Both mortars activated with CaCl1, VA50-CaCl1 and CVA45-BFS5-CaCl1 achieved high strengths at 90 days under thermal curing, at around 52 MPa. Conversely, they also showed higher porosities and water absorptions than VA50 at 28 days (Table 7). These properties may have contributed to the higher shrinkage observed, as greater porosity would encourage water loss.

Moreover, the use of mineral additions, FA and BFS, contributed to reducing shrinkage and reduced the absorption capacity of the VA50 mortars. The VA50-BSF5 achieved the lowest porosities and water absorptions of all the mortars (Table 7).

When comparing the effect of curing methods on the evolution of shrinkage value over time, it was observed that, in general, the mortar cured under thermal conditions achieved lower shrinkage than the mortar cured in a humid chamber. However, VA50 and VA40-FA10 mortars did not follow this trend, which exhibited 8% and 11% greater shrinkage values, respectively, in thermal curing. On the other hand, it is noteworthy that CVA50 achieved the lowest shrinkage value under both curing regimes. Additionally, the CVA50 mortar cured under thermal regime showed 24% less shrinkage value than the mortar with standard curing after 90 days. Bondar et al. [78] found that concrete subjected to temperature curing reached lower drying shrinkage and also observed a similar reduction in shrinkage values for mortars made with calcined pozzolans and subjected to thermal curing.

Moreover, mortars activated with CaCl2 (1%), VA50-CaCl1 and CVA45-BFS5-CaCl1 achieved the highest shrinkage values alongside the OPC. This increase in shrinkage, attributed to the use of CaCl2, is primarily associated with an increase in mesoporosity, which implies a greater contribution from changes in the pore size distribution within the matrix [79]. However, the effect of these changes in pore size distribution is often mitigated by high-temperature curing due to enhanced stabilisation of the C-S-H/C-A-S-H phases. The CVA45-BFS5-CaCl1 mortar cured under thermal conditions exhibited 22% less shrinkage value after 90 days than that cured under standard conditions, after 90 days. Other studies have shown that concrete made with BFS and alkali-activated natural pozzolans and cured at ambient temperature exhibited high shrinkage values, even reaching more than 2000 με [77].

#### 4.2.6. Acid Attack

According to weight loss results (Figure 5), when mortars were immersed in HCl solution and cured at 70 °C, they suffered greater weight loss than those cured under standard conditions. However, mortars containing VA and cured at 70 °C exhibited less degradation than OPC. In addition, the CVA50, VA45-BFS5, and CaCl-CVA45-BFS5 mortars showed the lowest weight losses. Moreover, the activated mortars CVA50, VA45-BFS5, and CVA45-BFS5-CaCl1 mortars achieved the highest residual strength. Furthermore, the activated mortar cured in standard conditions achieved less weight loss and lower strength decrease than the VA50 mortar, except the VA50-CaCl1 mortar.

When the mortars cured at standard conditions were immersed in H2SO4 solution, the CVA40-L10 mortar demonstrated the highest resistance to H_2_SO_4_ attack, achieving better performance than the OPC mortar. However, Yankwa Djobo et al. [31] attribute this behaviour to the formation of gypsum as a secondary phase resulting from the reaction between sulphuric acid and the calcium present in the matrix. According to thermally cured samples, the VA50-CaCl1 and CVA45-BFS5-CaCl1 mortars even increased in weight, which could be explained by the formation of gypsum, which has very low solubility in water. This dense gypsum layer may act as a surface sealant, slowing the deterioration process [31,80]. However, the CVA45-BFS5-CaCl1 mortar achieved the highest strength loss. It was also observed that VA50-based mortars submerged in H_2_SO_4_ experienced significantly lower weight loss when subjected to thermal curing and demonstrated higher acid resistance compared to OPC. In addition, all activated mortars displayed lower strength losses than the control VA50 mortar, except for VA40-FA10.

The mortars exposed to nitric acid (HNO_3_) were the most severe of the three acidic environments, regardless of the curing method applied. This could be attributed to the fact that, in the case of an HNO_3_ attack, the calcium salt (calcium nitrate) formed is highly soluble in water, leading to greater mass loss [80]. In general, the Alkali activated VA-based mortars cured at 70 °C resulted in lower weight and strength losses than those cured in standard conditions, in contrast to OPC. In standard condition, all mortars experienced significant strength losses (approximately 40–70%), except for CVA40-L10 and CVA45-BFS5-CaCl1, which showed much lower compressive strength reductions (20%).

As expected, there was a correlation between weight loss and strength loss after being submitted to an acid attack for the three acids studied. It is noteworthy that the CVA45-BFS5-CaCl1 and CVA40-L10 mortars, cured in thermal and standard conditions, showed smaller strength decreases.

According to the literature, it has been reported that mortars with equal proportions of VA and limestone powder, exposed to 6% H_2_SO_4_ for 90 days, exhibited a strength loss of 32%. However, as the replacement with natural pozzolan increased, the strength loss in the mortar decreased, reaching a strength loss of 10%. The low strength decrease in these mortars, and, consequently, the high resistance to acid, was attributed to the presence of high Si/Al and Ca/Si ratios in the C-A-S-H and N-A-S-H products, which resulted in pore-filling effects within the microstructure [49]. This behaviour could be related to the CVA40-L10 mortar, which achieved a strength decrease of only 25% in both curing conditions, significantly lower than the rest of the mortars, and is associated with the excellent mechanical performance observed.

In other studies, alkali-activated VA mortars incorporating FA exposed to 4% H_2_SO_4_, experienced a strength loss of 22% [81]. The authors attributed this behaviour to weaker bonds between the binders and the aggregate and decalcification and depolymerisation reactions. The formation of gypsum was also detected in the mortars from this study. These findings could linked to the VA50-CaCl1 and CVA45-BFS5-CaCl1 mortars, which showed strength losses despite showing weight increases.

## 5. Conclusions

The following conclusions can be drawn from the evaluation of the properties of mortars made with 50% VA as a replacement for OPC, subjected to different activation strategies and three curing procedures: standard condition and thermal (40 °C and 70 °C for 3 days, followed by standard condition).

### 5.1. Compressive Strength

- The thermal activation of VA, CVA50, achieved higher compressive strength than non-calcined VA50 at all tested ages and for all curing methods. The 70 °C curing for 3 days was the most effective regime for initial strength condition, achieving up to 45 MPa. In addition, at 90 days, it also achieved an adequate strength of 52 MPa. Under standard curing, the compressive strength gain of CVA50 was very consistent between 19 and 30% higher than that of VA50.
- Alkali-activated VA mortars using 2% of Na_2_SiO_3_ improved early-age compressive strength at 7 days, reaching 39–41 MPa when an initial 3 days 70 °C cured process was applied (OPC achieved 45.3 MPa); however, the strength increase development was low, achieving up to 43 MPa at 28 days.
- Alkali-activated VA mortars using 1% CaCl_2_ were the most efficient. The VA50-CaCl1 mortar, cured in standard condition or under an initial 3-day 70 °C curing process, reached the best compressive strength, achieving up to 40 MPa, 47 MPa and 52 MPa at 7, 28 and 90 days, respectively.
- Alkali-activated VA mortars using Na_2_CO_3_ did not achieve the strength obtained by the control VA mortar (VA50).
- The mortars produced using corrective agents L, FA, and BFS, in 10%, 10% and 5%, respectively, achieved up to 33% at 7 days and 46% at 28 days higher strength than the VA50 mortar. Although at 90 days, the obtained strength values were similar to those at 28 days, the VA40-FA10 and VA45-BFS5 achieved up to 44MPa and 46.8 MPa after curing in standard conditions and applied at the initial 3 days at 70 °C, respectively.
- The mixed-activated mortar, CVA45-BFS5-CaCl1, exhibited the highest compressive strengths at both 7 and 28 days, 47.4 MPa and 50.4 MPa, respectively, after submission of the thermal curing process, higher strengths than that of VA50 (up to 39 MPa). At 90 days, this mortar with standard curing achieved the highest strength of 56.3 MPa, surpassing VA50 by 32% (42.6 MPa) and reaching 88% of the strength of OPC (64.3 MPa).
- The activated VA50 mortar exposed to the thermal curing regime achieved the highest strength at 28 days. XRD and SEM-EDX results confirmed the following:
  - The microstructure of the activated VA mixtures had a higher presence of C-S-H and C-A-S-H phases. Gismondine and strätlingite were identified as pozzolanic reaction products, with gismondine being more prevalent in systems subjected to thermal activation. Additionally, the inclusion of CaCl_2_ as an activator promoted the formation of Friedel’s salt, which contributed to the high early-age strengths observed in these mixtures.

### 5.2. Drying Shrinkage

- The CVA mortar achieved the lowest shrinkage value in both curing processes, with −400 με and −600 με, respectively, being the only ones to show shrinkage lower than that of the VA50 mortar (−720 με). Thermal curing generally reduced drying shrinkage in comparison to standard curing, except for the VA50 and VA40-FA10 mortars. After being submitted to an initial 3 days 70 °C curing process, all the activated VA achieved a lower shrinkage value than that of VA50 mortar, which reached −800 με, except for the CaCl1-VA50-BFS10 mortar, which exhibited a 6% higher shrinkage value. The OPC achieved a −1100 με value after the standard curing process.

### 5.3. Acid-Attack Resistance

- When immersed in HCl, the VA mortars submitted to an initial 3 days at 70 °C curing process suffered higher weight loss and decreased strength than those submitted to standard curing conditions. In contrast, the VA mortars submitted to an initial 3 days at 70 °C curing process suffered a lower loss when they were immersed in H_2_SO_4_ and HNO_3_.
- The VA mortar achieved the lowest resistance to HNO_3_ attack, with up to 10% weight loss reduction, regardless of the curing method applied. The mixed activation mortar (CVA45-BFS5-CaCl1) demonstrated superior acid resistance under both curing conditions compared to other activation strategies. Furthermore, thermal curing enabled all mortars to achieve greater resistance than OPC mortar against all three acids studied, irrespective of the acid immersion.

The activation of VA through calcination, alkaline activation (using 1% CaCl_2_), the use of corrective agents (FA, BFS and L), and the mixed activation contributed to improving the mechanical, physical and durability properties of mortar produced using 50% of VA in replacement of OPC. The thermal curing at 70 °C for 3 days improved the early age compressive strength, the drying shrinkage and the acid resistance results of the 50% VA mortars. The CaCl1-VA50-BFS10 (with mixed activation methods) mortar submitted to an initial 3 days at 70 °C curing process showed similar compressive strength to OPC and higher resistance to acid attack. However, there was slightly increased porosity and drying shrinkage.

Future research should focus on evaluating the industrial-scale applicability and economic feasibility of activated volcanic ash-based mortar, including potential CO_2_ emission reductions and cost benefits compared to conventional OPC systems.

## Figures and Tables

**Table 1:** Chemical composition and LOI, in %, of the ordinary Portland cement (OPC), volcanic ash (VA), lime (L), ground-granulated blast-furnace slag (BFS), and fly ash (FA).

(%)	SiO_2_	Al_2_O_3_	CaO	Fe_2_O_3_	MgO	SO_3_	Na_2_O	K_2_O	Otros	LOI
OPC	19.4	4.2	63.5	3.4	1.4	3.0	0.12	0.53	-	3.7
VA	60.15	16.51	6.30	6.22	3.26	0.03	3.62	1.17	0.94	1.81
L	1.32	0.66	88.8	0.26	2.2	-	-	-	-	-
BFS	35.9	10.6	42.4	0.3	6.6	1.8	-	0.4	-	0.7
FA	58.4	21.6	2.3	7.3	1.9	0.2	-	0.9	-	3.1

**Table 2:** Composition and dosage of materials in grams (g) for mortar mixtures.

Mortar ID	OPC (g)	VA (g)	CVA (g)	L (g)	FA (g)	BFS (g)	NaSi (g)	CaCl (g)	NaCO (g)	FA (g)	Water (g)	SP (g)
OPC	500									1375	242	
VA50	250	250								1375	242	
CVA50	250	250	250							1375	242	
VA50-NaSi1	250	250					15			1375	233	0.2
VA50-NaSi1.5	250	250					22			1375	228	0.1
VA50-NaSi2	250	250					29			1375	223	0.6
VA50-NaSi3	250	250					44			1375	213	0.6
VA50-CaCl1	250	250						5		1375	242	
VA50-CaCl2	250	250						10		1375	242	
VA50-CaCl3	250	250						15		1375	242	
VA50-CaCl4	250	250						20		1375	242	
VA50-NaCO1	250	250							5	1375	242	
VA50-NaCO2	250	250							10	1375	242	
VA50-NaCO3	250	250							15	1375	242	
VA45-FA5	250	225			25					1375	242	
VA40-FA10	250	200			50					1375	242	
VA35-FA15	250	175			75					1375	242	
VA45-BFS5	250	225				25				1375	242	
VA40-BFS10	250	200				50				1375	242	
VA35-BFS15	250	175				75				1375	242	
CVA40-L10	250		200	50						1375	242	0.3
CVA45-BFS5-CaCl1	250		225			25		5		1375	242	

**Table 3:** Compressive strength results at 7 and 28 days (Phase 1) from all mortar mixtures under standard curing conditions; 90-day compressive strength results from those exhibiting superior mechanical performance at 7 and 28 days (Phase 2) and standard deviations of all results.

Mortar ID— St Curing	7 Days		28 Days		90 Days	
	CS (MPa)	St Dev (MPa)	CS (MPa)	St Dev (MPa)	CS (MPa)	St Dev (MPa)
OPC	45.29	0.81	56.55	2.25	64.26	1.76
VA50	25.75	1.58	31.77	3.42	42.59	0.89
CVA50	30.54	0.44	41.20	3.23	53.23	2.02
VA50-NaSi1	23.92	1.27	36.37	0.50		
VA50-NaSi1.5	22.00	1.91	30.67	4.18		
VA50-NaSi2	27.02	2.02	33.74	3.37		
VA50-NaSi3	25.84	0.59	31.54	1.10		
VA50-CaCl1	30.46	0.52	41.76	1.67	47.89	2.33
VA50-CaCl2	25.69	0.53	38.68	0.41		
VA50-CaCl3	23.15	1.86	34.58	0.54		
VA50-CaCl4	20.49	0.92	30.02	1.42		
VA50-NaCO1	23.37	0.93	33.19	3.64		
VA50-NaCO2	24.41	1.30	29.83	0.62		
VA50-NaCO3	16.72	0.67	22.95	1.00		
VA45-FA5	25.76	1.83	29.03	0.59		
VA40-FA10	29.74	0.04	38.78	1.19	54.78	3.04
VA35-FA15	30.21	2.14	39.62	0.12		
VA45-BFS5	30.00	1.25	44.38	1.65	51.65	0.94
VA40-BFS10	30.77	0.38	43.61	0.12		
VA35-BFS15	29.99	3.06	46.48	0.28		
CVA40-L10	32.86	1.61	40.21	1.48	49.10	0.24
CVA45-BFS5-CaCl1	29.39	1.65	42.78	0.78	56.27	0.79

**Table 4:** Compressive strength results at 7 and 28 days (Phase 1) from all mortar mixtures under thermal (40° for 3 days) curing conditions and standard deviations of all results.

Mortar ID—40 °C 3-Day Curing	7 Days		28 Days	
	CS (MPa)	St Dev (MPa)	CS (MPa)	St Dev (MPa)
OPC	43.81	1.73	56.62	1.73
VA50	35.47	0.44	39.12	2.24
CVA50	37.85	0.31	41.02	1.71
VA50-NaSi1	24.59	1.89	36.64	0.91
VA50-NaSi1.5	26.21	0.54	29.75	0.75
VA50-NaSi2	39.04	0.62	40.83	0.52
VA50-NaSi3	29.43	1.55	29.61	0.74
VA50-CaCl1	35.64	1.12	42.76	2.61
VA50-CaCl2	36.48	0.60	42.47	0.45
VA50-CaCl3	27.10	1.39	37.35	0.70
VA50-CaCl4	32.15	1.36	30.17	0.46
VA50-NaCO1	27.55	0.87	34.03	0.77
VA50-NaCO2	26.11	0.52	29.25	1.70
VA50-NaCO3	20.40	0.50	27.03	0.43
VA45-FA5	36.82	3.34	37.04	1.44
VA40-FA10	36.89	0.97	43.08	1.36
VA35-FA15	35.71	3.37	40.31	1.15
VA45-BFS5	40.19	0.42	46.23	2.36
VA40-BFS10	39.37	0.32	43.99	4.15
VA35-BFS15	42.76	0.92	46.14	2.22
CVA45-BFS5-CaCl1	38.80	1.80	48.74	2.78

**Table 5:** Compressive strength results at 7 and 28 days (Phase 1) from all mortar mixtures under thermal (70° for 3 days) curing conditions; 90-day compressive strength results from those exhibiting superior mechanical performance at 7 and 28 days (Phase 2) and standard deviations of all results.

Mortar ID—70 °C 3-Day Curing	7 Days		28 Days		90 Days	
	CS (MPa)	St Dev (MPa)	CS (MPa)	St Dev (MPa)	CS (MPa)	St Dev (MPa)
OPC	41.44	0.39	50.33	1.14	59.15	3.70
VA50	33.41	0.85	39.92	1.20	45.96	0.81
CVA50	43.60	0.16	45.08	1.68	52.41	1.55
VA50-NaSi1	33.90	1.04	41.60	1.38		
VA50-NaSi1.5	29.94	1.68	37.49	1.69		
VA50-NaSi2	41.00	2.90	42.85	0.87		
VA50-NaSi3	31.17	1.32	30.31	0.65		
VA50-CaCl1	39.95	3.83	46.96	2.47	52.34	1.61
VA50-CaCl2	35.33	1.04	42.15	0.53		
VA50-CaCl3	25.93	2.05	34.69	1.52		
VA50-CaCl4	27.64	2.10	30.48	0.45		
VA50-NaCO1	33.57	0.94	37.65	2.48		
VA50-NaCO2	29.94	2.41	31.77	1.22		
VA50-NaCO3	25.04	0.56	27.93	1.46		
VA45-FA5	40.78	3.12	45.60	1.85		
VA40-FA10	40.13	0.69	46.80	0.80	47.17	0.89
VA35-FA15	38.92	0.09	39.36	1.88		
VA45-BFS5	40.71	1.07	46.83	0.88	49.27	1.03
VA40-BFS10	43.84	3.04	45.17	1.08		
VA35-BFS15	44.27	2.64	46.24	1.69		
CVA40-L10	36.30	2.42	41.13	4.18	47.21	1.07
CVA45-BFS5-CaCl1	47.36	0.36	50.38	1.02	51.65	1.81

**Table 6:** Initial and final setting-time results from those mortars exhibiting superior mechanical performance.

Mortar ID	Setting Time	
	Initial (min)	Final (min)
OPC	140	190
VA50	165	205
CVA50	165	220
VA50-CaCl1	135	195
VA40-FA10	185	230
VA45-BFS5	150	195
CVA40-L10	155	210
CVA45-BFS5-CaCl1	135	195
ASTM C595-14	45	420 (max)

**Table 7:** Dry density, water absorption, and porosity results at 28 days from those mortars exhibiting superior mechanical performance.

Mortar ID	Standard Curing			70 °C 3-Day Curing		
	ρ (g/cm^3^)	WA (%)	P (%)	ρ (g/cm^3^)	WA (%)	P (%)
OPC	2.13	7.09	15.14			
VA50	2.08	7.53	15.66	2.11	6.59	13.89
CVA50	2.09	7.28	15.22	2.11	5.79	12.23
VA50-CaCl1	2.07	7.44	15.41	2.08	7.09	14.74
VA40-FA10	2.11	6.57	13.85	2.09	7.2	15.04
VA45-BFS5	2.11	6.48	13.68	2.11	5.58	11.76
CVA40-L10	2.07	7.3	15.07	2.10	5.66	11.87
CVA45-BFS5-CaCl1	2.09	6.26	13.07	2.06	8.49	17.48

**Table 8:** Residual compressive strength results from mortars cured for 28 days and subjected to acid (HCl, H_2_SO_4_ and HNO_3_) immersions for 90 days and compressive strength variation after acid immersion (compared to 28-day compressive strength described in Table 3, Table 4 and Table 5).

	Standard Curing						70 °C 3-Day Curing					
	HCl		H_2_SO_4_		HNO_3_		HCl		H_2_SO_4_		HNO_3_	
	σ_acid_ (MPa)	σ_loss_ (%)	σ_acid_ (MPa)	σ_loss_ (%)	σ_acid_ (MPa)	σ_loss_ (%)	σ_acid_ (MPa)	σ_loss_ (%)	σ_acid_ (MPa)	σ_loss_ (%)	σ_acid_ (MPa)	σ_loss_ (%)
OPC	51.28	−9.32	36.23	−35.93	15.53	−72.54	15.76	−68.69	14.4	−71.39	11.82	−76.52
VA50	20.85	−34.36	13.43	−57.72	11.56	−63.61	17.54	−56.06	17.4	−56.41	11.35	−71.56
CVA50	28.42	−31.01	14.52	−64.75	19.47	−52.74	38.21	−15.23	29.26	−35.09	26.01	−42.30
VA50-CaCl1	19.99	−52.13	13.5	−67.67	10	−76.05	23.03	−50.95	27.88	−40.62	14.13	−69.91
VA40-FA10	27.54	−28.97	12.98	−66.52	17.77	−54.17	22.56	−51.79	18.99	−59.42	19.36	−58.63
VA45-BFS5	47.45	−8.13	14.9	−71.15	25.32	−50.98	34.69	−25.92	22.66	−51.61	24.75	−47.14
CVA40-L10	46.23	−5.85	36.54	−25.58	39.62	−19.31	29.79	−27.57	36.99	−10.07	24.74	−39.85
CVA45-BFS5-CaCl1	53.4	−5.10	24.96	−55.64	44.24	−21.38	35.58	−29.37	35.46	−29.61	24.72	−50.93

## Data Availability

No data were used for the research described in the article.
